# Automated synthesis of [^89^Zr]ZrCl_4_, [^89^Zr]ZrDFOSquaramide-bisPh(PSMA) and [^89^Zr]ZrDFOSquaramide-TATE

**DOI:** 10.1186/s41181-024-00270-2

**Published:** 2024-05-08

**Authors:** Asif Noor, Peter D. Roselt, Emily R. McGowan, Stan Poniger, Michael P. Wheatcroft, Paul S. Donnelly

**Affiliations:** 1 School of Chemistry and Bio21 Molecular Science and Biotechnology Institute, University of Melbourne, Parkville, VIC 3010 Australia; 2 Department of Radiopharmaceutical Sciences, Cancer Imaging, The Peter MacCallum Cancer Centre, Melbourne, VIC 3000 Australia; 3 Sir Peter MacCallum Department of Oncology, The University of Melbourne, Melbourne, VIC 3010 Australia; 4 iPHASE Technologies Pty Ltd., Rowville, VIC 3178 Australia; 5 Telix Pharmaceuticals Limited, Suite 401, 55 Flemington Road, North Melbourne, VIC 3051 Australia

**Keywords:** Zirconium-89, Radiopharmaceuticals, Automated synthesis, [^89^Zr]ZrCl_4_

## Abstract

**Background:**

Automated [^89^Zr]Zr-radiolabeling processes have the potential to streamline the production of [^89^Zr]Zr-labelled PET imaging agents. Most radiolabeling protocols use [^89^Zr][Zr(ox)_4_]^4−^ as the starting material and oxalate is removed after radiolabeling. In some instances, radiolabeling with [^89^Zr]ZrCl_4_ as starting material gives better radiochemical yields at lower reaction temperatures. In this work, a fully-automated process for production of [^89^Zr]ZrCl_4_ is reported and its use for the synthesis of [^89^Zr]ZrDFOSq-bisPhPSMA and [^89^Zr]ZrDFOSq-TATE.

**Results:**

A simple automated process for the isolation of [^89^Zr]ZrCl_4_ by trapping [^89^Zr][Zr(ox)_4_]^4−^ on a bicarbonate-activated strong anion exchange cartridge followed by elution with 0.1 M HCl in 1 M NaCl was developed. [^89^Zr]ZrCl_4_ was routinely recovered from [^89^Zr][Zr(ox)_4_]^4−^ in > 95% yield in mildly acidic solution of 0.1 M HCl in 1 M NaCl using a fully-automated process. The [^89^Zr]ZrCl_4_ was neutralized with sodium acetate buffer (0.25 M) removing the requirement for cumbersome manual neutralization with strong base. The mixture of [^89^Zr]ZrCl_4_ was used for direct automated radiolabeling reactions to produce [^89^Zr]Zr-DFOSquaramide-bisPhPSMA and [^89^Zr]ZrDFOSquaramide-TATE in 80–90% over all RCY in > 95% RCP.

**Conclusions:**

This method for the production of [^89^Zr]ZrCl_4_ does not require removal of HCl by evaporation making this process relatively fast and efficient. The fully automated procedures for the production of [^89^Zr]ZrCl_4_ and its use in radiolabeling are well suited to support the centralized and standardized manufacture of multiple dose preparations of zirconium-89 based radiopharmaceuticals.

**Supplementary Information:**

The online version contains supplementary material available at 10.1186/s41181-024-00270-2.

## Background

The radioactive half-life of zirconium-89 and relatively low translation energy of the positron emission from zirconium-89 (*t*_1/2_ = 78.4 h, *β*^+^  = 22.3%, *E*_ave_(β^+^) = 396.9 keV) have led to the isotope becoming the radionuclide of choice for radiolabelling of monoclonal antibodies to make antibody-based tracers for Positron Emission Tomography (PET) (Verel et al. 2003; Feo et al. 2022; Yoon et al. 2020; Heskamp et al. 2017; Dongen et al. 2021; McInnes et al. 2017; Jauw et al. 2019). The high molecular weight and interactions mediated by the Fc portion of antibodies (Fc = Fraction crystallisable) lead to radiolabelled antibodies taking days to clear from the blood pool and localise in the target tissue. PET tracers that rely on peptides or small molecules to achieve selectivity accumulate in target tissue more rapidly and are consequently often radiolabelled with radionuclides with shorter radioactive half-lives such as gallium-68 (*t*_1/2_ = 68 min) or fluorine-18 (*t*_1/2_ = 109.8 min). PET tracers that are radiolabelled with gallium-68 are currently playing a major role in diagnosis by PET imaging, but the short radioactive half-life of gallium-68 often requires the radiopharmaceutical to be synthesised on-site. There is increased interest in the potential to use zirconium-89 for peptide-based tracers where the longer radioactive half-life of zirconium-89 would make centralized manufacture of PET tracers to a certified good manufacturing process (GMP) standard feasible. In principle, the distribution of GMP PET tracers from a centralized manufacturer to hospitals would greatly increase the number of clinical sites that could perform PET scans although the additional radiation dose associated with zirconium-89 needs to be considered. The longer radioactive half-life also offers the possibility of imaging at later time points where reduced background signal can lead to the identification of lesions that are not present on scans using gallium-68 labelled tracers (Felix et al. 2022; Rosar et al. 2023).

A common route for the preparation of zirconium-89 is proton bombardment of yttrium-89 in a ^89^Y(*p,n*)^89^Zr reaction. The zirconium-89 radionuclide is then purified from the ^89^Y starting material by resin that has been functionalised with hydroxamate functional groups that form strong interactions with [^89^Zr]Zr^IV^. Elution of the resin with oxalic acid (0.05–1 M) leads to excellent recovery of [^89^Zr]Zr^IV^ where the oxalic acid deprotonates and serves as a dianionic bidentate ligand to form complexes best formulated as [[^89^Zr]Zr(ox)_4_]^4−^. The isolated [[^89^Zr]Zr(ox)_4_]^4−^ solution is well suited for both transportation and complexation reactions presumably through transfer chelation to ligands such as desferrioxamine (H_3_DFO) and H_3_DFO-antibody conjugates. Although the solutions of [[^89^Zr]Zr(ox)_4_]^4−^ in oxalic acid can be used for directing labelling of H_3_DFO antibody conjugates it is essential that oxalic acid/oxalate is removed before administration as it is highly toxic. The removal of oxalate/oxalic acid is often achieved by size-exclusion chromatography for antibody conjugates which adds an extra manipulation with radioactive materials. Separation of oxalate from [^89^Zr]Zr^IV^ labelled peptide-based or peptidomimetic-based probes with lower molecular weight is more challenging. For radiolabelling both antibodies and small molecules/peptides with [^89^Zr]Zr^IV^ it is possible that removing oxalate before addition of the biomolecule could be a better approach and certain radiolabelling reactions have been demonstrated to proceed better when [^89^Zr]ZrCl_4_ is used as starting material rather than [[^89^Zr]Zr(ox)_4_]^4−^. It is possible to isolate [^89^Zr]ZrCl_4_ by loading [[^89^Zr]Zr(ox)_4_]^4−^ onto a strong anion exchange cartridge in the chloride form followed by elution with aqueous HCl (1 M) (Holland et al. 2009). The HCl is then removed by boiling the eluate at 110 °C under a continuous stream of argon and it is then reconstituted in 0.9% saline or 0.1 M HCl for radiolabelling. Removing HCl by evaporation increases the time required for synthetic preparations and can lead to loss of activity due to the formation of aerosols potentially increasing radiation exposure, and contamination. A promising alternative to the solid target ^89^Y(*p,n*)^89^Zr methods for the production of [^89^Zr]Zr is the proton bombardment of aqueous solutions of [^89^Y]YCl_3_ and [^89^Y](NO_3_)_3_ (Pandey et al. 2014). A potential advantage of this liquid target approach is that it avoids the solid target processing steps (do Carmo et al. 2020).

**Fig. 1:**
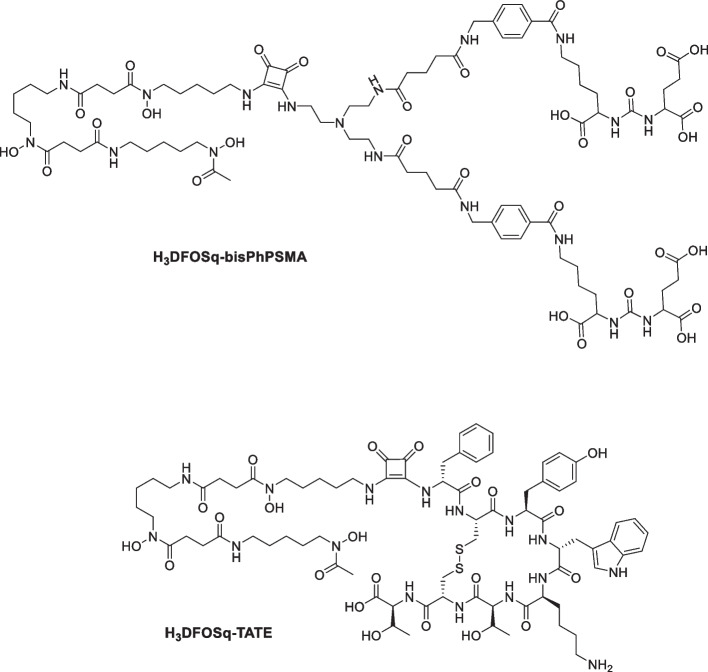
Chemical structures of H_3_DFOSq-bisPhPSMA and H_3_DFOSq-TATE

In this work, we describe a new processes for the synthesis of [^89^Zr]ZrCl_4_ from [^89^Zr][Zr(ox)_4_]^4−^ using a bicarbonate-activated polystyrene-based strong anion exchange cartridge. This processes directly produces solutions of [^89^Zr]ZrCl_4_ in mixture of dilute HCl (0.1 M) in aqueous NaCl (1 M). When working with radioactivity it is important to keep the amount of radiation used as low as is possible (ALARA) and to minimize radiation exposure to those involved in manufacture whilst synthesizing pure, sterile products in a reproducible fashion to support centralized manufacture and distribution to multiple clinical sites. With these goals in mind we translated the chemistry for the production of [^89^Zr]ZrCl_4_ to an automated procedure using a commercially available iPHASE MultiSyn radiosynthesizer. We have then extended the approach to use [^89^Zr]ZrCl_4_ produced on the synthesizer to prepare a small molecule and peptide tracers, [^89^Zr]ZrDFOSq-bisPSMA (Fig. 1, a bivalent inhibitor of prostate specific membrane antigen) (Noor et al. 2020), [^89^Zr]ZrDFOSq-TATE (Fig. 1, a somatostatin subtype-2 receptor-targeting peptide) (Noor et al. 2021). This work builds on previous contributions for automated production of [^89^Zr]Zr^IV^-based radiopharmaceuticals that used [^89^Zr][Zr(ox)_4_]^4−^ as a starting material (Dongen et al. 2019; Wichmann et al. 2023).

## Results

### *Synthesis of [*^*89*^*Zr]ZrCl*_*4*_

**Fig. 2:**
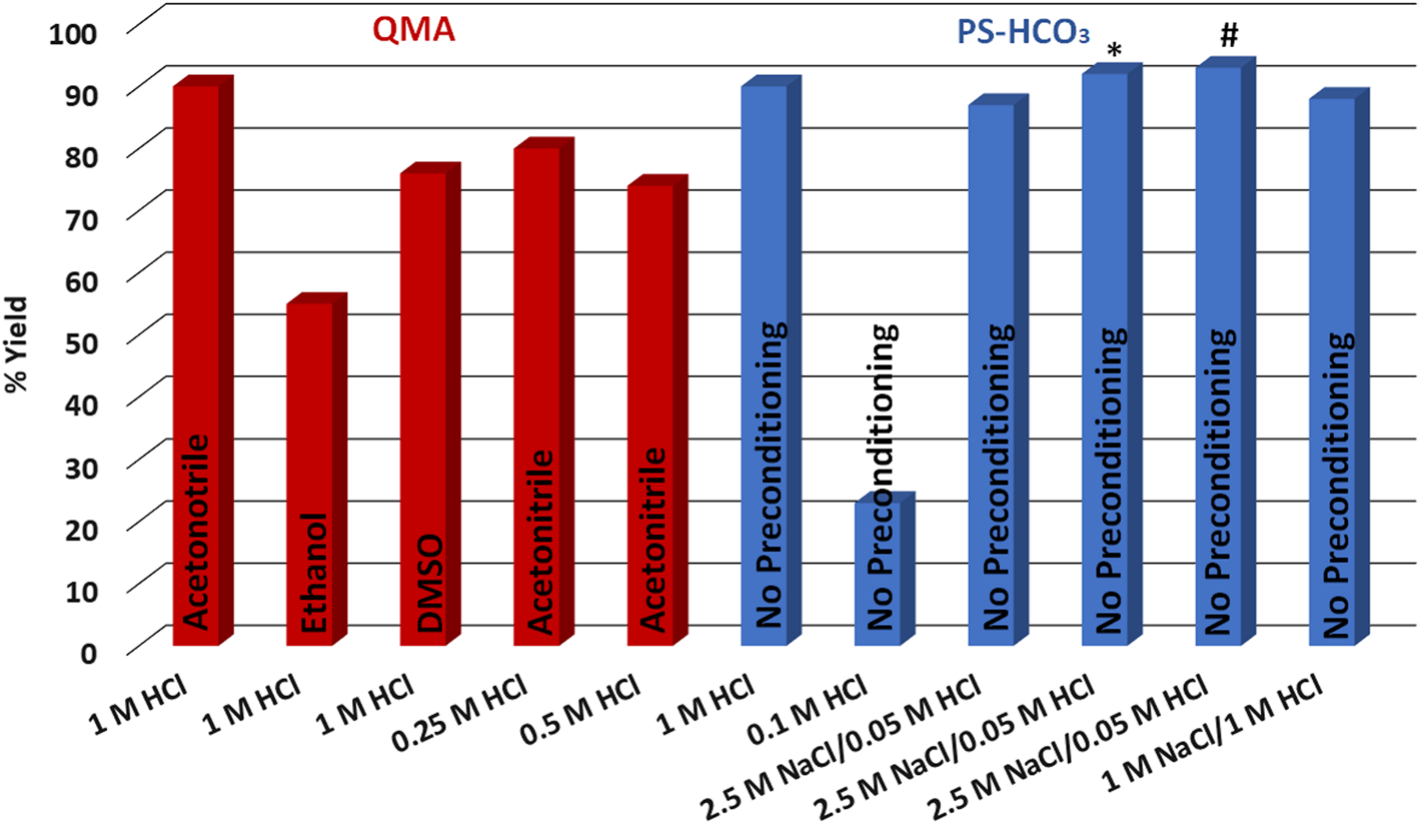
Manual production of [^89^Zr]ZrCl_4_ from QMA and PS-HCO_3_ cartridges under different conditions, with different pre-conditioning solvents stated on each entry, [[^89^Zr][Zr(ox)_4_]^4−^ in 0.05 M oxalic acid was used other than *0.5 M, #1.0 M oxalic acid

One goal of this study was to develop automated standardized, reproducible syntheses of [^89^Zr]ZrCl_4_, [^89^Zr]ZrDFOSq-bisPSMA and [^89^Zr]ZrDFOSq-TATE that could form the basis of procedures used to prepare tracers suitable for clinical studies. It was deemed preferable to remove oxalate/oxalic acid and produce [^89^Zr]ZrCl_4_ which was then used for the radiolabeling reactions. We first repeated published procedures for the preparation of [^89^Zr]ZrCl_4_ using a Quaternary Methyl Ammonium (QMA) strong anion exchange cartridge (Waters Sep-pak QMA, with a quaternary amine functional group in the chloride form) that was pre-conditioned with acetonitrile (Pandya et al. 2019). Consistent with literature procedure, maximum recovery of [^89^Zr]ZrCl_4_ required elution of the cartridge with 1 M HCl (~ 90%, Additional file 1: Figure S2, Table S1). Acetonitrile is considered a ‘Class 2’ solvent by both the European Medicines Agency and the U.S. Food and Drug Administraion where use “should be limited” (Q3C(R8) Impurities 2021; ICH guideline Q3C (R8) 2022; Constable et al. 2007; Grodowska and Parczewski 2010), so pre-conditioning the column with either ethanol or dimethylsulfoxide was investigated but both resulted in lower recovered yields of [^89^Zr]ZrCl_4_ (55%-76%, Fig. 2, Table S1) as did elution with 0.25 M HCl (~ 80%, Fig. 2, Additional file 1: Table S1).

An alternative anion exchange cartridge, a functionalized crosslinked polystyrene-divinylbenzene polymer in the bicarbonate form (PS-HCO_3_ SAX cartridge, Huayi Isotopes) was also investigated. This column is marketed for separation of fluorine-18 where the manufacturer’s instructions state that this column does not require pre-conditioning with organic solvents prior to use as it is pre-treated with ethanol (Class 3 solvent) and plugged tightly at both ends to prevent contamination and manufactured according to Good Manufacturing (GMP) requirements. Following application of [[^89^Zr][Zr(ox)_4_]^4−^ to the cartridge (in either 1 M or 0.05 M oxalic acid mixtures) [^89^Zr]ZrCl_4_ was eluted with HCl (1 M) in > 90% recovered yield (Fig. 2, Table S1). Radiolabelling of DFO-derivatives requires neutralisation of 1 M hydrochloric acid with a strong base such as sodium carbonate. The efficiency of elution with mixtures of aqueous sodium chloride and dilute hydrochloric acid (0.1 M and 0.05 M) was investigated to investigate if eluting with an increased chloride ion concentration but with a lower concentration of acid was possible. The combination of hydrochloric acid (0.05 M) with sodium chloride solution (2.5 M NaCl) resulted in excellent recovery of ~ 92%. This is slighter lower recovery than when 1 M hydrochloric acid is used, but the recovery of [^89^Zr]ZrCl_4_ in a dilute (0.05 M) hydrochloric acid solution has the significant advantage that this eluent can be readily buffered with sodium acetate buffer and does not require the addition of a stronger base (sodium carbonate) (Fig. 2).

### *Synthesis of [*^*89*^*Zr]ZrDFOSq-bisPhPSMA from [*^*89*^*Zr]ZrCl*_*4*_

The [^89^Zr]ZrCl_4_ eluent (0.1 M HCl/1 M NaCl) was used to prepare [^89^Zr]ZrDFOSq-bisPhPSMA that we published previously (Noor et al. 2020, 2021), with the omission of the ‘neutralization’ step with sodium carbonate and C18 SPE purification. Addition of [^89^Zr]ZrCl_4_ eluent (100 MBq, 0.1 M HCl/1 M NaCl) to a mixture of the respective ligand (100 ng) in sodium acetate (0.25 M) buffer and heating the reaction mixtures to 75 °C for 15 min allowed isolation of each compound in > 95% radiochemical yield and purity that does not require any SPE purification. This new methodology allowed the use of a significantly lower peptide concentration (100 ng/MBq compared with 2.0 µg/MBq) than our initial method increasing the molar activity to 18.8 GBq/μmol at the end of synthesis. Another advantage of using [^89^Zr]ZrCl_4_ and dissolving the ligands in ethanol was that the final product does not contain either oxalate or dimethyl sulfoxide which were removed in our initial synthesis by a final purification through a C18 solid-phase extraction cartridge.

### *Automated production of [*^*89*^*Zr]ZrCl*_*4*_*, [*^*89*^*Zr]ZrDFOSq-bisPhPSMA and [*^*89*^*Zr]ZrDFOSq-TATE*

**Fig. 3:**
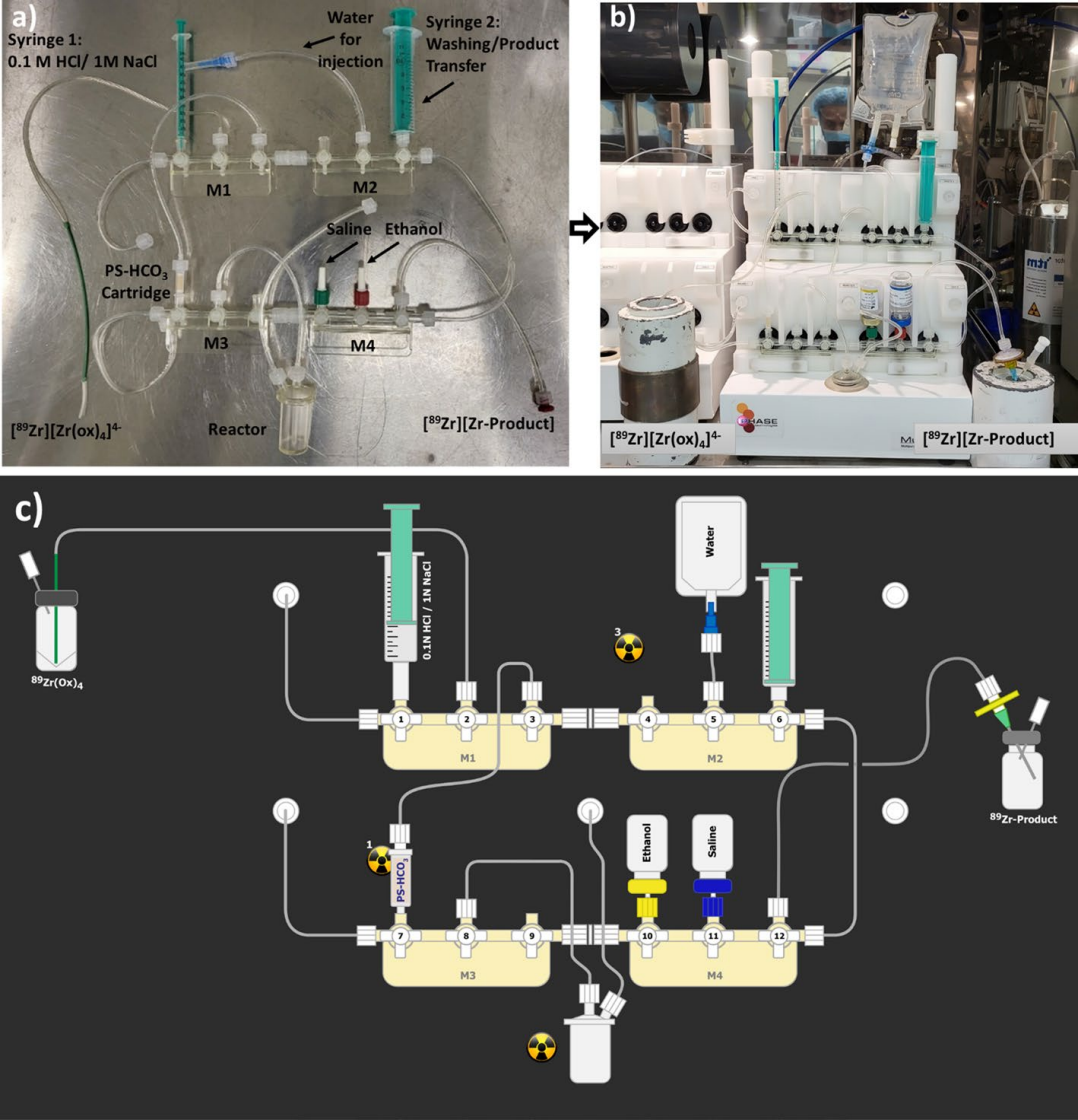
**a** MultiSyn ZrCl disposable cassette configuration assembled and **b** installed cassette on an iPHASE Multisyn for the production of [^89^Zr]ZrDFOSq-bisPSMA/TATE, **c** schematic diagram of the production process

The new synthetic methodology for the preparation of [^89^Zr]ZrDFOSq-bisPhPSMA and [^89^Zr]ZrDFOSq-TATE directly from [^89^Zr]ZrCl_4_ was next translated to a fully automated procedure using a commercial iPHASE MultiSyn synthesis module using disposable cassettes (Wichmann et al. 2023, 2021). An iPhase zirconoium-89 radiolabelling disposable cassette (MSH-300) was modified and configured according to Fig. 3a then mounted on the Multisyn module (Fig. 3b). The iPHASE Multisyn programmed sequence was downloaded on the control software and prompts were followed to commence the synthesis. In the first step excellent trapping (> 95%) [^89^Zr]Zr^IV^ was observed on the PS-HCO_3_ cartridge and high recoveries (90%) of [^89^Zr]ZrCl_4_ were achieved as observed on the built-in radiodetectors on manifold 3. The cartridge was eluted at a slower flow rate with a mixture of NaCl (1.0 M) and HCl (0.1 M) over 5 min to provide maximum recovery. The [^89^Zr]ZrCl_4_ (~ 150–1000 MBq) was directly eluted into the reaction vial containing H_3_DFOSq-bisPhPSMA dissolved in a mixture of aqueous sodium acetate buffer (0.25 M), ethanol (10%) and ascorbic acid (0.05%). The transfers of liquids were controlled through remote control software by opening and closing the gas pressure/vacuum valves or by using the syringe drivers. The reaction vessel was then heated at 75 °C for 15 min resulting in the synthesis of [^89^Zr]ZrDFOSq-bisPhPSMA in an overall > 80% radiochemical yield, based on [[^89^Zr]Zr(ox)_4_]^4−^, with a radiochemical purity of > 95% determined by radio-TLC (Figure S1) and radio-HPLC (Figure S2) with a final step that includes in-line sterile filtration (Table S2). The final sterile filtered product was collected in 25 mL evacuated vials, a total volume of 12–14 mL in 0.9% w/v of saline was obtained as a clear colorless solution with no visible particles and a pH of 5–6. No residual oxalate detected using QuantiQuik™ Oxalate Quick Test Strips.

Adapting the procedure developed for the synthesis of [^89^Zr]ZrDFOSq-bisPhPSMA to [^89^Zr]ZrDFOsq-TATE resulted in radiochemical yields of ~ 60% with ~ 30% of the radioactivity being retained in the reaction vial, tubing, syringe and the sterile filter. [^89^Zr]ZrDFOsq-TATE is less soluble than [^89^Zr]ZrDFOsq-bisPhPSMA in the aqueous sodium acetate (0.25 M), ethanol (10%) and ascorbic acid (0.05%) mixture. Increasing the ethanol concentration to 20% in the reaction vessel increased the yield to 67%. Adding an addition washing step with an ethanol vial (2 mL) to position #10 (Fig. 3c) to rinse the reaction vial and the cassette followed by an additional transfer of the product mixture in 0.9% w/v saline allowed isolation of [^89^Zr]ZrDFOsq-TATE in > 85 ± 3% radiochemical yields based on [^89^Zr][Zr(ox)_4_)]^4−^ with > 95% radiochemical purity (Additional file 1: Figures S3 and S4, Table S3). The final product (12–14 mL) was filtered through a sterile filter into evacuated vials such that final formulation contained 0.9% saline containing ≤ 20% ethanol and 0.05% ascorbic acid. This material would be sufficient for multiple administrations and could be further diluted to ensure the ethanol concentration is ≤ 10% (Serdons et al. 2008).

The new automated procedure produced similar yields and purity when two different commercial sources of [[^89^Zr][Zr(ox)_4_]^4−^ that are provided with different concentration of oxalic acid (1 M and 0.05 M) and with [[^89^Zr][Zr(ox)_4_]^4−^ within 1 day of production as well as 4 and 10 days post-production were used (Additional file 1: Tables S2–S3). Similar radiochemical yields were also obtained when two different sources of H_3_DFOsq-bisPhPSMA and H_3_DFOsq-TATE were used (one prepared in-house and one prepared by a commercial GMP manufacturer, Supporting Information, Table S2-S3). The procedures described here produced similar results with radioactivity ranging from 150 MBq to 1.02 GBq. As a final validation at least seven independent syntheses of each tracer were completed. Each independent synthesis was assessed by five parameters, radiochemical purity (by HPLC and radioTLC), radiochemical yield, visual appearance and pH (Additional file 1: Tables S2–S3). Each independent synthesis resulted in > 95% radiochemical purity at end of synthesis, as analyzed by both radio-HPLC and radio-iTLC, with radiochemical yields of 80–90%.

## Discussion

In this work we developed a method for the synthesis of [^89^Zr]ZrCl_4_ that used an ion exchange cartridge (PS-HCO_3_ SAX) that is supplied in the bicarbonate form. This cartridge does not require pre-conditioning with acetonitrile, a significant advantage when compared to cartridges that use QMA resin in the chloride form. Following application of [[^89^Zr]Zr^IV^(ox)_4_]^4−^ in oxalic acid to PS-HCO_3_ SAX cartridges, elution with a mixture of hydrochloric acid (0.05 M)/ sodium chloride (2.5 M) allowed isolation of [^89^Zr]ZrCl_4_ with recovery efficiencies of ~ 92%. The relatively low concentration of acid used allows the pH to be buffered with sodium acetate and does not require the addition of a stronger base (sodium carbonate). This pH neutralisation step is cumbersome and often performed manually by careful adjustment of pH to minimise the formation of zirconium hydroxide, and colloidal species (McInnes et al. 2017; Aja et al. 1995; Hu et al. 2013). It is possible that the weakly basic HCO_3_^−^ counterion, which would react with hydrochloric acid to give carbon dioxide and water, coupled with the different ion-pairing and ion-exchange properties of HCO_3_^−^ contribute to efficient elution with relatively low concentrations of hydrochloric acid/sodium chloride. In a separate investigation that evaluated the efficiency of eluting of [^18^F]F^−^ from QMA cartridges with diaryliodonium salts the HCO_3_^−^ form of the QMA cartridge out performed all other anions tested (Maisonial-Besset et al. 2018).

The PS-HCO_3_ SAX cartridge can be easily incorporated into an automated radiopharmaceutical production synthesiser and we developed an automated process using a process for producing [^89^Zr]ZrCl_4_ that was adapted to a commercial iPHASE MultiSyn synthesis module using disposable cassettes. Excellent trapping of [^89^Zr]Zr^IV^ (> 95%) was observed on the PS-HCO_3_ cartridge and [^89^Zr]ZrCl_4_ could be recovered with ~ 90% efficiency when eluted with a mixture of NaCl (1.0 M) and HCl (0.1 M). This mixture can be used directly for radiolabeling reactions. For example, the [^89^Zr]ZrCl_4_ can be directly eluted into reaction vials pre-loaded with H_3_DFO conjugates such as H_3_DFOSq-bisPhPSMA dissolved in a aqueous buffer for direct radiolabelling. Our automated process allowed isolation of [^89^Zr]ZrDFOSq-bisPhPSMA in ~ 90% yield in greater than 97% RCP without the need of C18 SPE purification. For the automated synthesis of [^89^Zr]ZrDFOSq-TATE the radiochemical yields was optimized by the addition of higher quantities of ethanol and with an additional ethanol rinse step. The ethanol concentration can be reduced to ~ 10% by dilution with saline (Serdons et al. 2008). The automated processes reported here are amenable to scale-up for centralized multiple-dose preparation and validation to current good manufacturing practice (cGMP) standards. The automated methodology for the production of [^89^Zr]ZrCl_4_ presented here can also be used as precursor in the formation of [^89^Zr]ZrDFOSq-antibody conjugates and this will be reported in a separate publication.

## Conclusion

Application of [^89^Zr][Zr(ox)]^4−^ to a PS-HCO_3_ SAX cartridge followed by elution with a mixture of 0.1 M hydrochloric acid and sodium chloride allowed isolation of oxalate free [^89^Zr]ZrCl_4_. This method does not require removal of HCl by evaporation making this process relatively fast and efficient. This process has also been adapted to an automated procedure on a commercial synthesizer. The mixture of [^89^Zr]ZrCl_4_ in dilute acid can be neutralized with sodium acetate buffer allowing for direct reactions with ligands and has been used in a fully automated process for the synthesis of [^89^Zr]ZrDFOSq-bisPhPSMA and [^89^Zr]ZrDFOSq-TATE in high radiochemical yields with high radiochemical purity. The fully automated procedures for the production of [^89^Zr]ZrCl_4_ and its use in radiolabeling are well suited to support the centralized manufacture of multiple dose preparations of zirconium-89 based radiopharmaceuticals.

## Methods

### *Manual [*^*89*^*Zr]ZrCl*_*4*_* production using QMA cartridge and radiolabelling of H*_*3*_*DFOSq-bisPSMA*

[[^89^Zr][Zr(ox)_4_]^4−^ was purchased from Austin Health in 0.05 M Oxalic acid. Waters Sep-Pak Accell Plus QMA Plus Light Cartridge, 130 mg sorbent per cartridge, 37–55 µm was used to convert [[^89^Zr][Zr(ox)_4_]^4−^ to [^89^Zr]ZrCl_4_. The cartridge was activated using 6 mL of acetonitrile, 10 mL of 0.9% saline and 10 mL Milliq Water. [[^89^Zr][Zr(ox)_4_]^4−^ (100–200 µL in 0.05 M oxalic acid, 50 -150 MBq) was loaded onto a preactivated QMA cartridge. The cartridge was then washed with water (50 mL) to remove oxalic acid and then the activity was eluted as [^89^Zr]ZrCl_4_with 400 µL of 1.0 M HCl in a typical recovery of 80–90%. [^89^Zr]ZrCl_4_ (10–100 μL, 5 vol. equiv., 10–100 MBq, in 1 M HCl,) was neutralised to pH 4–5 with sodium acetate (3 M) then ethanol (1 vol. equiv.) was added to make final labelling mixture containing 20–25% ethanol and 0.6 M sodium acetate. H_3_DFOSq-bisPSMA (0.4 µg/MBq, in 1:1 MQ:ethanol) was added and the reaction mixture was agitated at 75 °C for 15 min. The reaction mixture was analysed by radio-HPLC. The traces showed > 95% radiochemical yield with a radiochemical purity of > 95%.

### *Manual [*^*89*^*Zr]ZrCl*_*4*_* production using READI-CLING™ PS-HCO*_*3*_* SAX cartridge*

READI-CLING™ PS-HCO_3_ SAX cartridge was obtained from Huayi Isotopes. The cartridge was conditioned with MilliQ water (10 mL) then [[^89^Zr][Zr(ox)_4_]^4−^ solution in 0.05–1 M oxalic acid was passed through the cartridge. The cartridge was then washed with water (50 mL) to remove oxalic acid and then the activity was eluted as [^89^Zr]ZrCl_4_with 1 N NaCl/0.1 M HCl (1 mL) in > 90% radiochemical yield.

### General method for Automated production of [^89^Zr]ZrDFOSq-bisPSMA/TATE

The manufacturing room was prepared in accordance with facility standard operating procedures. The reagents were either sourced commercially or freshly prepared in the lab, 0.1 M HCl solution in 1 M sodium chloride was drawn in a sterile 1 mL syringe provided with the disposable cassette, stored capped with sterile needle until required. H_3_DFOSq-bisPSMA/TATE precursors were dissolved in ethanol/water (1:1) to make a 5 mg/mL solution then added a volume of precursor solution corresponding to the mass of precursor required for the radiolabelling to a 1.2 mL 0.25 M sodium acetate vial. To the same vial, additional ethanol was added to make a final ethanol concentration of 20% (v/v) and 2.5% Na-gentisate (w/v). The disposable cassette MSH-3000 was modified and assembled as shown in Fig. 3a then mounted on the MultiSyn module Fig. 3b. The iPHASE Multisyn module was then switched on and the sequence scheme was loaded and prompts followed to perform initial leak checks. Following the sequence prompts, the 1 mL syringe containing 0.1 M hydrochloric acid in 1 M sodium chloride solution was mounted at position 1 (manifold 1), the vial containing 10 mL saline for injection was mounted at position 11 (manifold 4), 2 mL ethanol in a vial was mounted at position 10 (manifold 4), 100 mL water for injection bag was placed on a spike at position 5 (manifold 2), precursor solution was loaded into the center port of the reactor, the pre-prepared 15 mL sterile product collection vial in a shielded transport container was connect with the product delivery line at position 12 (manifold 4), and finally the calibrated sample of [[^89^Zr][Zr(ox)_4_]^4−^ in oxalic acid in a 15 mL Falcon tube was connected to the device via a PEEK needle inlet at position 2 (manifold 1). The hot cell was closed and the sequence prompts were followed to commence radiosynthesis. The synthesis was completed in approximately 30 min. The product collection vial was removed and final product volume was calculated (12–15 mL), the product activity was recorded using a calibrated dose calibrator to calculate radiochemical yield (80–92%), and the product underwent QC analysis by radio-HPLC (95%), radio-TLC (> 95%), pH (5–7) and visual appearance testing (colorless and particulate free). The products were found to be stable up to 6 days from EOS when stored at room temperature.

### Supplementary Information

**Additional file 1**. Tables S1–S3; Synthesis S1 and S2; Figures S1–S4.

## Data Availability

All data generated or analysed during this study are included in this published article [and its Additional file 1].
