# Supplementary material for: Automated synthesis of [89Zr]ZrCl4, [89Zr]ZrDFOSquaramide-bisPh(PSMA) and [89Zr]ZrDFOSquaramide-TATE
Source: EJNMMI Radiopharm Chem. 2024 May 8;9:39. doi: 10.1186/s41181-024-00270-2 (PMC11078908; doi:10.1186/s41181-024-00270-2)

**Supporting Information**

**Automated Synthesis of [^89^Zr]ZrCl_4_, [^89^Zr]ZrDFOSquaramide-bisPh(PSMA) and [^89^Zr]ZrDFOSquaramide-TATE**

Asif Noor,*^a^ Peter D. Roselt,^b,c^ Emily R. McGowan,^a^ Stan Poniger^d^, Michael P. Wheatcroft^e^ and Paul S. Donnelly*^a^

^a^School of Chemistry and Bio21 Molecular Science and Biotechnology Institute University of Melbourne, Parkville, Victoria 3010, Australia.

^b^Department of Radiopharmaceutical Sciences, Cancer Imaging, The Peter MacCallum Cancer Centre, Victoria 3000, Australia;

^c^Sir Peter MacCallum Department of Oncology, The University of Melbourne, Victoria 3010, Australia;

^d^iPHASE technologies Pty Ltd, Rowville, VIC 3178, Australia

^e^Telix Pharmaceuticals Limited, Suite 401, 55 Flemington Road, North Melbourne, VIC 3051, Australia.

Corresponding authors: asif.noor@unimelb.edu.au and [pauld@unimelb.edu.au](mailto:pauld@unimelb.edu.au)

**Contents**

- Table S1 Manual ^89^Zr-Cl_4_ production from QMA and PS-HCO_3_ cartridges
- Synthesis S1: Synthesis and pre-validation of [^89^Zr]ZrDFOSq-bisPSMA
- Figure S1 Representative example of Radio-TLC of [^89^Zr]ZrDFOSq-bisPSMA
- Figure S2 Representative example of Radio-HPLC of [^89^Zr]ZrDFOSq-bisPSMA
- Table S2 Pre-Validation matrix for [^89^Zr]ZrDFOSq-bisPSMA
- Synthesis S2: and pre-validation of [^89^Zr]ZrDFOSq-TATE
- Figure S3 Representative example of Radio-TLC of [^89^Zr]ZrDFOSq-TATE
- Figure S4 Representative example of Radio-HPLC of [^89^Zr]ZrDFOSq-TATE
- Table S3 Pre-Validation matrix for [^89^Zr]ZrDFOSq-TATE

**Table S1.** Manual ^89^Zr-Cl_4_ production from QMA and PS-HCO_3_ cartridges

| Cartridge | Activating Solvent | ^89^Zr-(Ox)_2_  (MBq) | Oxalic Acid  conc. (M) | Eluting Solvent | % Recovery |
| --- | --- | --- | --- | --- | --- |
| QMA | Acetonitrile | 50-150 | 0.05 | 1 | 90-93% |
| QMA | Ethanol | 64 | 0.05 | 1 | 55% |
| QMA | DMSO | 33 | 0.05 | 1 | 76% |
| QMA | Acetonitrile | 40 | 0.05 | 0.25 | 80% |
| QMA | Acetonitrile | 58 | 0.05 | 0.5 | 74% |
| PS-HCO_3_ | Nil | 40 | 0.05 | 1 M HCl | 90% |
| PS-HCO_3_ | Nil | 40 | 0.05 | 0.1 M HCl | 23% |
| PS-HCO_3_ | Nil | 45 | 0.05 | 2.5 M NaCl/0.05 M HCl | 87% |
| PS-HCO_3_ | Nil | 64 | 0.5 | 2.5 M NaCl/0.05 M HCl | 92% |
| PS-HCO_3_ | Nil | 40 | 1 | 2.5 M NaCl/0.05 M HCl | 93% |
| PS-HCO_3_ | Nil | 100 | 0.05 | 1 M NaCl/0.1 M HCl | 88% |

**Synthesis S1: Synthesis and pre-validation of [^89^Zr]ZrDFOSq-bisPSMA**

Following the general procedure (Table S2, entry 7), 0.1 M HCl solution in 1M sodium chloride was drawn in a sterile 1 mL syringe provided with the disposable cassette, stored capped with sterile needle until required. Dissolved DFOSq-bisPSMA (10 mg) precursor in ethanol/water (1:1, 2 mL) to make a 5 mg/mL solution then added a 200 μL of precursor solution to the 1.2 mL 0.25M sodium acetate vial. To the same vial, added additional 300 µL ethanol to make a final ethanol concenntaration of 18-20% and 200 μL of 2.5% Na-gentisate. The disposable cassette MSH-3000 was modilfied and assemble as shown in Figure 3a then mounted on MultiSyn module Figure 3b. The iphase Multisyn module was then switched on and the sequence scheme was loaded and followed the prompt to performed intinal leaks checks. Following the sequence prompts, the 1 mL syringe containing 0.1M hydrochloric acid in 1M sodium chloride solution was mounted at position 1 (manifold 1), and the vial containing 10 mL saline for injection at position 11 (manifold 4) and 2 mL ethanol in vial at position 10 (manifold 4), 100 mL water for injection bag onto position 5 (manifold 2), precursor solution into the centre port of the reactor, the pre-prepared 15 mL sterile product collection vial in a shielded transport container and connect with the product delivery line at position 12 (manifold 4) and finally the [^89^Zr]Zr-oxalate in oxalic acid (1020 MBq, 440 μL) with the PEEK needle inlet at position 2 (manifold 1). The hot cell was closed and the sequence prompts were followed to commence radiosynthesis. The synthesis was completed in approx 30 min then the production collection vial was removed and final product volume was calculated (approx 12 mL), the product activity was recorded using a calibrated dose calibrator to calculate yield radiochemical yield (90%) and product was tested for QC analysis by radio-HPLC (95%), radio-TLC (>95%), pH (5-7) and visual apperance parameters (colorless no visible particles). The products was found stable upto 6 days EOS when stored at room temperature.

**Figure S1.** Representative example of Radio-TLC of [^89^Zr]ZrDFOSq-bisPSMA, product obtained from Table S2 entry 7

**Figure S2.** Representative example of Radio-HPLC of [^89^Zr]ZrDFOSq-bisPSMA, product obtained from Table S2 entry 7

Table S2 Pre-Validation matrix for [^89^Zr]Zr-DFOSq-bisPSMA


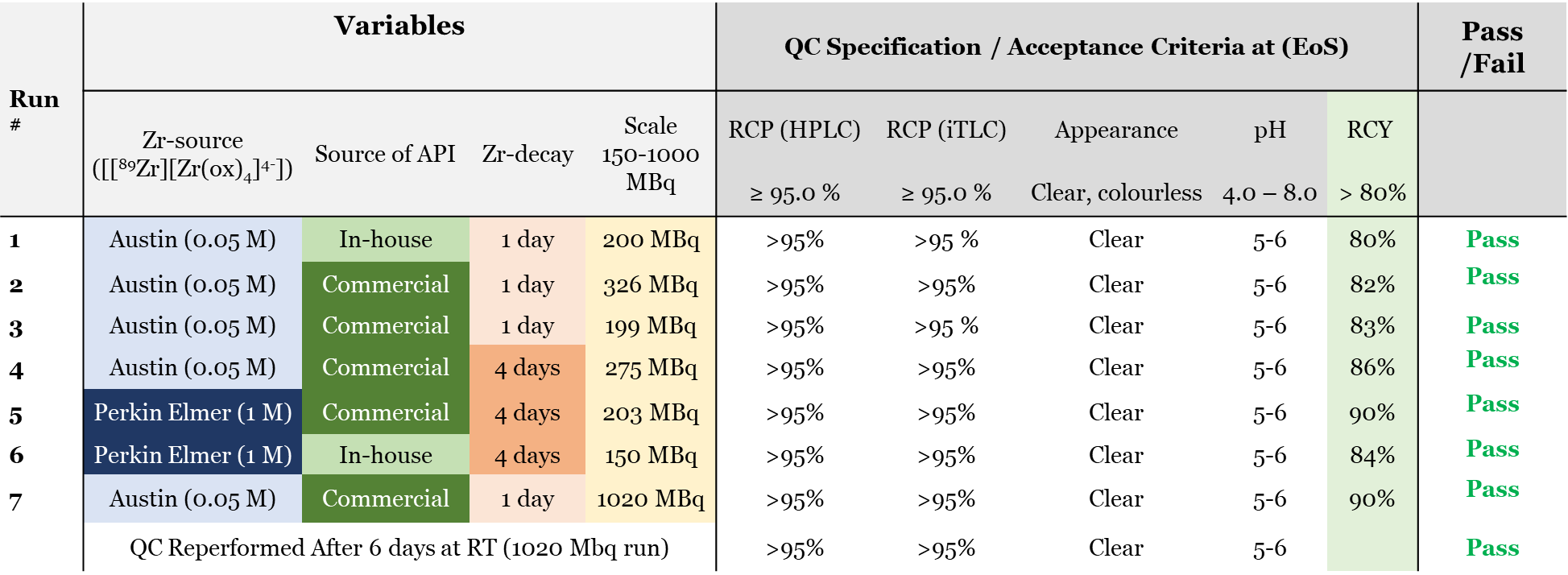


**Synthesis 2: Synthesis and pre-validation of [^89^Zr]ZrDFOSq-TATE**

Following the general procedure (Table S3, entry 9), 0.1 M HCl solution in 1M sodium chloride was drawn in a sterile 1 mL syringe provided with the disposable cassette, stored capped with sterile needle until required. Dissolved DFOSq-TATE (10 mg) precursor in ethanol/water (1:1, 2 mL) to make a 5 mg/mL solution then added a 205 μL of precursor solution to the 1.2 mL 0.25M sodium acetate vial. To the same vial, added additional 300 µL ethanol to make a final ethanol concentaration of 18-20% and 200 μL of 2.5% Na-gentisate. The disposable cassette MSH-3000 was modilfied and assemble as shown in Figure 3a then mounted on MultiSyn module Figure 3b. The iphase Multisyn module was then switched on and the sequence scheme was loaded and followed the prompt to performed intinal leaks checks. Following the sequence prompts, the 1 mL syringe containing 0.1M hydrochloric acid in 1M sodium chloride solution was mounted at position 1 (manifold 1), and the vial containing 10 mL saline for injection at position 11 (manifold 4) and 2 mL ethanol in vial at position 10 (manifold 4), 100 mL water for injection bag onto position 5 (manifold 2), precursor solution into the centre port of the reactor, the pre-prepared 15 mL sterile product collection vial in a shielded transport container and connect with the product delivery line at position 12 (manifold 4) and finally the [^89^Zr]Zr-oxalate in oxalic acid (1030 MBq, 345 μL) with the PEEK needle inlet at position 2 (manifold 1). The hot cell was closed and followed the sequence prompts to commence radiosynthesis. The synthesis was completed in approx 30 min then the production collection vial was removed and final product volume was calculated (12 mL), the product activity was recorded using a calibrated dose calibrator to calculate yield radiochemical yield (90%) and product was tested for QC analysis by radio-HPLC (95%), radio-TLC (>95%), pH (5-7) and visual apperance parameters (colorless no visible particles). The products was found stable upto 6 days EOS when stored at room temperature.

**Figure S3.** Representative example of Radio-TLC of [^89^Zr]ZrDFOSq-TATE, product obtained from Table S2 entry 7

**Figure S4.** Representative example of Radio-HPLC of [^89^Zr]ZrDFOSq-TATE, product obtained from Table S2 entry 7

**Table S3.** Pre-Validation matrix for [^89^Zr]ZrDFOSq-TATE


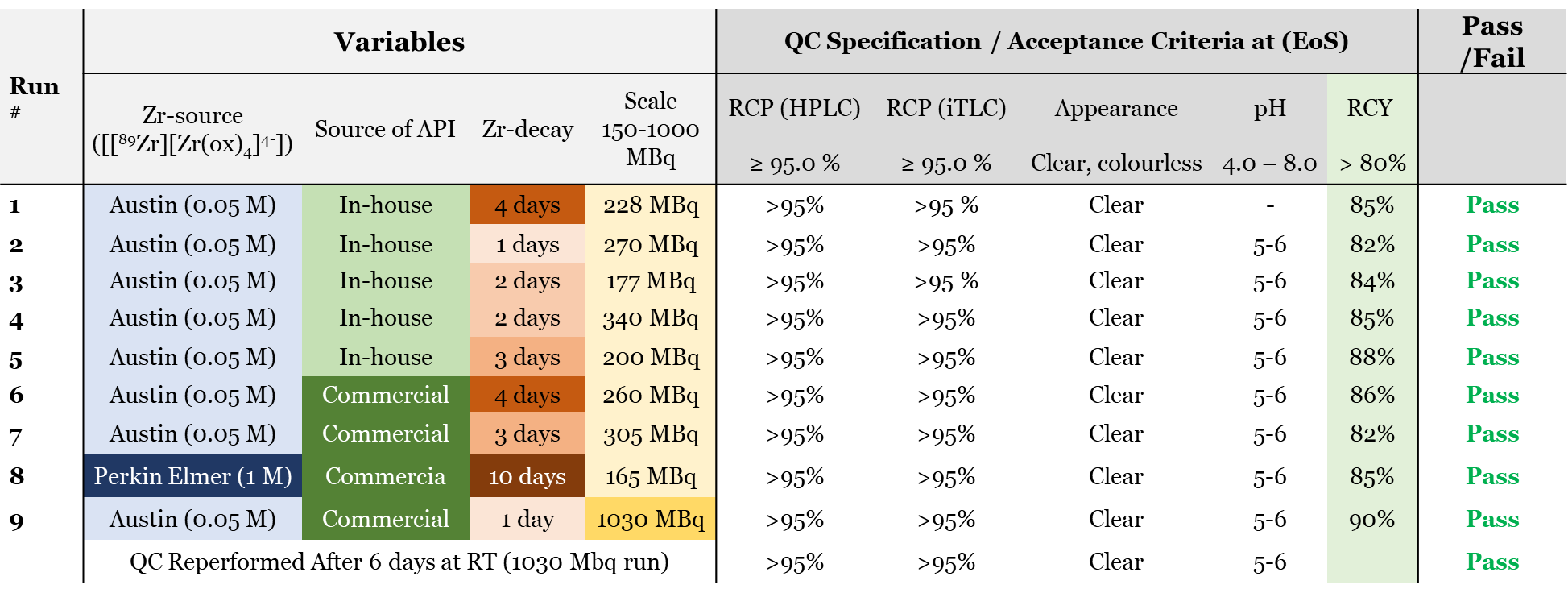

Supplement: Supplementary file 1 — Additional file 1. Tables S1–S3; Synthesis S1 and S2; Figures S1–S4. [file 41181_2024_270_MOESM1_ESM.docx]
